# Uncovering dimensions of the impact of blockchain technology in supply chain management

**DOI:** 10.1007/s12063-022-00273-9

**Published:** 2022-06-11

**Authors:** Ulpan Tokkozhina, Ana Lucia Martins, Joao C. Ferreira

**Affiliations:** 1 Business Research Unit (BRU-IUL), Lisbon, Portugal; 2 Instituto Universitário de Lisboa (ISCTE-IUL), 1649-026 Lisbon, Portugal; 3 Inov Inesc Inovação—Instituto de Novas Tecnologias, 1000-029 Lisbon, Portugal; 4 Information Sciences and Technologies and Architecture Research Centre (ISTAR-IUL), Lisbon, Portugal; 5 Molde University College — Specialised University in Logistics, NO-6410 Molde, Norway

**Keywords:** Blockchain technology, Supply chain management, Dimensions of impact, Advantages, Disadvantages, Constraints

## Abstract

Supply chains around the globe are faced with difficulties and disruptions due to the worldwide pandemic situation and digital solutions are needed. There is significant research interest in the implementation of blockchain technology (BCT) for supply chain management (SCM). A challenge that remains is analyzing the interactions of BCT in different areas of SCM. This study aims to identify the influential dimensions of the impact of BCT adoption in SCM and to discuss the synergetic and counter-synergetic effects between these dimensions. Advantages, disadvantages, and constraints of adopting BCT in the SCM context are explored through a systematic literature review, which provides the foundation for identifying the dimensions of impact. The interactions between these dimensions are conceptually discussed. This study introduces three dimensions of the impact of implementing BCT in SCM: ‘operations and processes’, ‘supply chain relationships’, and ‘innovation and data access’. These dimensions are interrelated and have overlapping areas within them, which leads to synergetic and counter-synergetic effects. The overlaps and synergies of the three dimensions of impact are illustrated, and the virtuous and vicious cycles of BCT adoption in SCM cases are highlighted. This study assists scholars and practitioners by clarifying the synergetic relationships within the dimensions of the impact of BCT in SCM and by providing considerations to prevent undesirable effects and expand desired ones.

## Introduction

Supply chains (SCs) face unprecedented challenges. The tendency to lengthen them (Chopra et al. 2021) increases their vulnerability in terms of information sharing, and trust poses difficulties in their management. To overcome this problem, SC managers actively seek disruptive solutions that bring innovativeness, efficiency, and effectiveness to their operations (Behnke and Janssen 2020). The global state of emergency due to the Covid-19 pandemic in 2020 highlighted the need for SC digitalization. The digitalization phenomenon is already shaping new realities and relationship models throughout the entire SC network (Queiroz and Fosso Wamba 2019). One of the most recognizable disruptive technologies for SC adoption is blockchain technology (BCT) (Queiroz et al. 2020).

Blockchain is defined as a ‘cryptographically guaranteed non-tamperable and unforgeable distributed decentralized ledger’ (Liu and Li 2020, p.2). BCT makes it possible for supply chain management (SCM) activities and operations to occur in a highly secure and transparent way, without the need for a central organization (Gonczol et al. 2020). Thus, it is a potential game-changer for supply chains (SCs) in terms of information exchange and verified transactions (Hackius and Petersen 2020). However, the adaptation blueprint of BCT still requires a more focused and comprehensive evaluation (Wong et al. 2020a), as well as an understanding of the critical requirements, issues, and challenges across the SC (Ghode et al. 2020b; Wang et al. 2020b) for successful BCT deployment.

Endeavours to systematize BCT in the SCM context have been undertaken by numerous scholars, with a focus on, i.e. application areas (Queiroz et al. 2019; Chang and Chen 2020; Duan et al. 2020), industrial contexts (Wang et al. 2019a; Idrees et al. 2021), concept definition (Fosso Wamba et al. 2020a, b), bibliometric analysis (Fosso Wamba et al. 2020a; Müßigmann et al. 2020), and article coding (Lim et al. 2021). Systematization has also been explored in the transport and logistics sector (Kummer et al. 2020; Pournader et al. 2020), food SC (Kayikci et al. 2020; Stranieri et al. 2021), and from information-sharing perspectives (Wan et al. 2020). The potential benefits, challenges, drivers, and barriers to BCT adoption in SCM have also been studied using empirical data (van Hoek 2019; Wang et al. 2019b; Stranieri et al. 2021) as well as in specific fields such as agriculture (Kamilaris et al. 2019), the textile and clothing industry (Agrawal et al. 2021) and modern military solutions (Saadiah and Rahayu 2021). A common feature of these wide-ranging attempts to study BCT in SC is a case-specific perspective.

There are as well more generalized reviews that were found in prior literature, that were not attaching findings to a specific area of application. Such studies were building comprehensive maps of separate BCT application topics and themes for supply chain performance (Mahyuni et al. 2020), indicating the most influential authors and publications in the area (Moosavi et al. 2021), separately summarizing enablers, benefits, and risks of BCT adoption (Karakas et al. 2021), conceptualizing stages and factors behind the adoption (Vu et al. 2021).

Extant literature reviews offer many contributions in specific sectors, e.g. food and agriculture SCs (Galvez et al. 2018; Lezoche et al. 2020; Sharma et al. 2021), or reviews of a mix of different technologies under the scope of Industry 4.0 (Bodkhe et al. 2020; Mistry et al. 2020). There as well were studies that grouped themes into clusters (Klerkx et al. 2019) and structured topics and trends into mind maps (Casino et al. 2019). Recently scholars were conducting bibliometric analysis of literature in the field (Moosavi et al. 2021; Tandon et al. 2021), in the context of operations management, mechanisms of a traceability feature of BCT were explored (Feng et al. 2020). Thematic analysis, identifying the orders of benefits and challenges domains of BCT adoption in food SC was as well performed (Chen et al. 2021). However, there is still a lack of a more holistic understanding of the ways in which BCT implementation impacts SCM and operations, outside of a specific application area or context, based on systematized data.

Among open issues of BCT exploration in SC context, such research gaps as suitability of BCT for specific projects, and a mechanism to choose a proper blockchain network that would suite the actual needs of the application was highlighted (Casino et al. 2019). The need of leading through emerging technologies adoption was mentioned (Klerkx et al. 2019), in order to enhance the benefits and alleviate potential negative effects. Research is needed to guide through decision-making process of the adoption of BCT for businesses (Frizzo-Barker et al. 2020). It is also necessary to investigate the societal, organizational, and environmental factors that affect BCT implementation in SCM (Tandon et al. 2021).

Therefore, the contribution of the present research goes beyond prior studies to provide a broader vision and holistic understanding of the existing features of BCT in the SCM context, grouping them into unified general dimensions, without limiting it further to a specific industry or area of application. Like this, it would contribute to gaps identified by previous scholars in the field. Against this backdrop, this research aims to create a systematized understanding of BCT in the SCM context. The goals of this study are threefold: (1) to explore the dimensions of the impact of BCT implementation in SCM; (2) to explore the synergies of these dimensions; and (3) to explore the virtuous and vicious cycles of the adoption of BCT use in SCM.

To achieve these goals, a systematization of the existing literature is pursued. Specifically, BCT features found in the literature are systematized, the operations management dimensions of the impact of BCT implementation in SCM are exposed, and the synergetic and counter-synergetic effects of these dimensions in terms of the advantages, disadvantages, and constraints of BCT implementation are discussed.

The remainder of this paper is organized as follows. Section 2 provides a literature review of the BCT phenomenon in the SC context, as well as current SCM challenges. Section 3 details the methodology adopted in this study. Section 4 provides a systematized understanding of BCT features in the SCM context, which are conveniently displayed in tables and clarifies the logic process underpinning the dimensions and their synergies. Section 5 discusses the theoretical and managerial contributions of this study and suggests areas to be addressed in future studies. Finally, Sect. 6 provides the conclusions of the study and critically identifies its limitations.

## Literature review

Today, supply chains are beginning to lose the necessary visibility across networks due to their length (Mubarik et al. 2021). SC visibility is the transparency to identify product transit status throughout and outside the supply chain (Roy 2021). It plays a crucial role in strengthening trust and collaboration among different stakeholders (Baah et al. 2021). Thus, to gain visibility for SCs, organizations must establish a way to share timely and reliable information across networks (Tapscott and Tapscott 2020).

Another major SCM focus of organizations is a long-term stability perspective: building confidence and reliance among SC participants (i.e., building trust) (Paluri and Mishal 2020). The long and complex nature of SC networks requires interactions between multiple entities, resulting in challenges in establishing trust among them (Doroudi et al. 2020). An increasing level of trust in the SC scale leads to risk reduction (Collier and Sarkis 2021) and more stable end-consumer behavior (Doroudi et al. 2020).

### Blockchain technology

Blockchain is a fully distributed and decentralized system that can capture and store cryptographically encoded data and transactions in a block manner, similar to a distributed ledger (Queiroz et al. 2019). The mechanism behind BCT then connects each block—which is identified with a unique hash code (a cryptographic algorithm that transforms a data input into a fixed-length irreversible output)—to a previous block, thus connecting all blocks into a chain (Queiroz et al. 2020; Wang et al. 2020c). This chain structure is the fundamental feature that ensures immutable and reliable data in the ledger, as each block holds details (the unique hash code) of the previous blocks’ content (Gonczol et al. 2020). Thus, BCT allows safe data exchange within the SC in a distributed manner (Wang et al. 2019b).

BCT brings traceability to SCs, allowing assets to be tracked and providing stakeholders with the visibility required to do so (Choi 2020; Wang et al. 2020b). Through BCT adoption, it is possible to reduce the incidence of unfair practices and counterfeits (Juma et al. 2019; Stranieri et al. 2021) and boost the overall efficiency of SC operations (Leng et al. 2018). Another important innovation that BCT entails is smart contracts, which are, essentially, ‘digital contracts that flow across enterprises’ (Kamble et al. 2019, p.2011), enabling the automation of processes (Nandi et al. 2020) because smart contracts are self-executable (Min 2019). Overall, BCT has the potential to manage resources efficiently and reduce inventory levels (Kamble et al. 2019), reduce SC risks through traceability features (Kshetri 2018; Montecchi et al. 2019), and optimize planning and forecasting decisions (Perboli et al. 2018).

### Blockchain benefits and challenges in the context of supply chain management

BCT also has attractive capabilities for SC participants; it can improve collaboration between entities (Mao et al. 2018), especially in untrusted environments (Liu and Li 2020), and reduce the bullwhip effect (Perboli et al. 2018). Increasing SC performance and data visibility (Hald and Kinra 2019) results in product quality compliance and improves trust among consumers (Ghode et al. 2020b; Nandi et al. 2020). To improve practitioners’ experience, BCT can, for example, be used together with other artificial intelligence technologies (Kayikci et al. 2020) and combined with Internet of Things (IoT) devices (Rejeb et al. 2019).

However, BCT has several disadvantages. Although BCT can provide traceability through digital records, it cannot ensure the real condition of physical products in transit (Yadav and Singh 2020) and is not always a suitable solution, depending on the cost of the product. For example, for lower-cost products, there is less need to thoroughly trace products (van Hoek 2019). Smart contracts are also considered difficult to implement if the coding of the contract is of poor quality, which usually leads to a series of problems throughout the SC (Cole et al. 2019). The lack of central authority within the SC is sometimes considered a disadvantage, especially when resolving disputes among SC entities (Wang et al. 2019a, b). Another major concern is an environmental one: BCT consumes excessive amounts of energy to conduct transaction-related activities (Öztürk and Yildizbaşi 2020; Yadav et al. 2020).

Some challenges still exist in modern SCM, including, e.g. relationship and trust issues between participants in complex SCs, considerations regarding firms’ social responsibilities, concerns over the visibility of operations that reveal the dynamics of supply processes, and issues related to exploiting SCM to influence consumers’ buying decisions and gain a competitive advantage (Cole et al. 2019; van Hoek 2020a, b; Rogerson and Parry 2020). BCT is considered a possible solution to these limitations of modern SCs; however, a rigorous academic investigation is still required to understand the extent to which BCT may create value for firms (Treiblmaier 2018).

Additionally, there are some practical constraints in the adoption of BCT in the SCM context. For example, a lack of a relevant legal framework (Sheel and Nath 2019) can lead to difficulties in convincing all stakeholders to adopt BCT (Duan et al. 2020). A lack of trust among SC network participants, national circumstances, and cultural differences can also result in different decisions during the adoption process (Kopyto et al. 2020; Queiroz et al. 2020; Wong et al. 2020a). Currently, most SCs are not yet ready for BCT adoption due to either the lack of an organized ecosystem (Kamble et al. 2019) or the novelty of the technology (Min 2019), as adoption of this technology is a complex process. Not only it requires appropriate infrastructure, but it also needs to overcome challenges related to existing regulatory and legal governance systems (Kamble et al. 2021).

BCT adoption poses constraints for supply chain participants in terms of coping with the innovative decentralized structure (Omar et al. 2020). Dimensions of BCT characteristics were introduced in the context of supply chain collaboration (Rejeb et al. 2021), manufacturing industry (Karamchandani et al. 2021), healthcare supply chains (Aich et al. 2021), and categorized key enablers of BCT accordingly to its aims and opportunities (Ali et al. 2021). However, to the best of our knowledge, a more holistic approach on BCT dimensions of impact and their intersections exploration in terms of supply chain implementation was not yet performed. A perceptible knowledge gap between managers of various organizations and their comprehension of BCT was revealed (Walsh et al. 2021), thus an intelligible representation of potential dimensions of impact and their interconnection would assist practitioners in terms of novel technology understanding and applications.

## Methodology

**Fig. 1:**
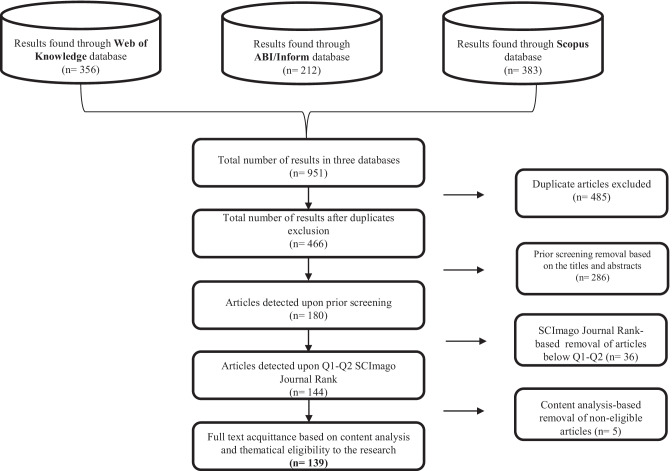
Literature selection process

To comprehensively address this study’s aims, a systematic literature review (SLR) was chosen as the most suitable methodology, as it enables following a pre-planned route to enable the most relevant existing literature to be chosen, evaluated, and analyzed to generate the study’s findings (Denyer and Tranfield 2009). For the SLR, this study followed the Preferred Reporting Items for Systematic Reviews and Meta-Analysis (PRISMA) checklist (Moher et al. 2009). Adherence to the PRISMA checklist ensures clarity in the review process for others and maintains a structured process, as it requires a transparent motion in the study selection and analysis processes (Saunders et al. 2012). As implied by the PRISMA checklist, for convenience, the literature selection process needs to be schematically presented in a flow diagram; Fig. 1 introduces the selection process conducted for this study.

Three academic databases were selected for this study: Web of Knowledge (WoS), ABI/Inform, and Scopus. These databases were chosen as trusted global platforms for academic searches and for their ability to provide top multidisciplinary journal and article results. The keywords for the search were ‘blockchain’ and ‘supply chain’; an advanced search function was used to collect articles with both keywords found in the title and abstract. The search was also limited to peer-reviewed articles written in English. All publications available up to January 2022 were included, without any limitations per year.

As can be seen in Fig. 1, the number of studies identified in each database was as follows: WoS (356), ABI/Inform (212), and Scopus (383). Thus, a total of 951 articles were identified. The first step was to exclude the duplicates, which resulted in 466 unique articles. The next stage was a prior screening of articles based on the titles and abstracts, to include articles focused on operations management, logistics, specific supply chain applications, and to avoid more technical articles that go beyond the scope of the following research. Examples of topics that did not meet the prior screening criteria include, but are not limited to: humanitarian SCs, supply chain finance, detailed technical descriptions of smart contracts and protocols, crypto-asset applications, energy delivery systems etc. It is important to note, that the research objects were not limited to any specific article types, due to the novelty of the topic, thus all studies, that were compliant with the screening criteria mentioned above, were included. The prior screening step resulted in 180 selected articles. Next, to increase the level of screening and classification, only articles from Q1 and Q2 academic journals (based on SCImago Journal Rank) were included, which resulted in a total of 144 articles. The full texts of these 144 articles were screened, and based on content analysis and eligibility to the study, 139 articles were selected for inclusion in this SLR.

Table 1 shows the exemplary list of journals and the number of publications that were found among the final selected set of 139 articles, to identify the most frequent journals in which the articles were published. The three leading journals are (1) the International Journal of Production Research, (2) IEEE Access and (3) the International Journal of Information Management. This sequence once again highlighted the multidisciplinary nature of the topic, given that academic articles were found in both SCM and production/logistics journals, as well as IT-focused journals. The types of articles also varied from more theoretical to technological applications. These 139 articles were then thoroughly and carefully analyzed to identify the advantages, disadvantages, and constraints (features) of BCT application to SCM, highlighted by the authors. These features were further classified based on meaning and content to ensure the integrity of the findings.

**Table 1:** Journals with the top number of published articles in the field of blockchain technology in supply chain management

**JOURNALS**	**NUMBER OF ARTICLES**
International Journal of Production Research	15
IEEE access	11
International Journal of Information Management	10
Supply Chain Management – An International Journal	8
International Journal of Production Economics	7
Sustainability (Switzerland)	7
Journal of Business Logistics	6
Future Internet	4
Computers and Industrial Engineering	4
Operations Management Research	3
Production Planning & Control	3
Resources Conservation and Recycling	3
Technological Forecasting and Social Change	3

## Dimensions of the impact of blockchain technology implementation in supply chain management

Overall, as can be seen in Tables 2, 3 and 4 the number of advantages of BCT use in SCM is greater than the disadvantages, and the number of constraints is also limited. The features identified in Tables 2, 3 and 4 are listed in order, from the most reported to the least reported.

**Table 2:** Advantages of blockchain technology in supply chain management context

**Advantages of BCT in SCM context**	**Authors**	**Explanation**
Transparency and traceability	Treiblmaier 2018; Leng et al. 2018; Mao et al. 2018; Min 2019; Fu and Zhu 2019; Juma et al. 2019; Kamble et al. 2019; Saberi et al. 2019; Montecchi et al. 2019; Wang et al. 2019a; Queiroz and Fosso Wamba 2019; Cole et al. 2019; Hald and Kinra 2019; Chang et al. 2019; Rejeb et al. 2019; O’Leary 2019; Azzi et al. 2019; Cui et al. 2019; Gurtu and Johny 2019; Wang et al. 2019b; Kamilaris et al. 2019; Schmidt and Wagner 2019; van Hoek 2019; Chang et al. 2020; Gonczol et al. 2020; Cha et al. 2020; Bai and Sarkis 2020; van Hoek 2020a; van Hoek 2020b; Durach et al. 2020; Öztürk and Yildizbaşi 2020; Sternberg et al. 2020; Choi 2020; Howson 2020; Rogerson and Parry 2020; Behnke and Janssen 2020; Di Vaio and Varriale 2020; Kamble et al. 2020; Liu and Li 2020; Tönnissen and Teuteberg 2020; Ghode et al. 2020b; Nandi et al. 2020; Casino et al. 2020; Kayikci et al. 2020; Wang et al. 2020a, b, c; Köhler and Pizzol 2020; De Giovanni 2020; Duan et al. 2020; Fosso Wamba et al. 2020a; Kopyto et al. 2020; Fosso Wamba et al. 2020b; Dutta et al. 2020; Wan et al. 2020; Shoaib et al. 2020; Xue et al. 2020; Sunny et al. 2020; Stranieri et al. 2021; Musamih et al. 2021; Lim et al. 2021; Ahmad et al. 2021; Kayikci et al. 2021; Menon and Jain 2021; Li et al. 2021a; Li et al. 2021b; Centobelli et al. 2021; Reddy et al. 2021; Yang et al. 2021	BCT transparency is increasing the visibility (Min 2019), thus enabling actions like tracing, recording and tracking (Kamble et al. 2019)
Cost reduction	Perboli et al. 2018; Kshetri 2018; Min 2019; Fu and Zhu 2019; Cui et al. 2019; Gurtu and Johny 2019; Montecchi et al. 2019; Queiroz et al. 2019; Fosso Wamba et al. 2020a; Wang et al. 2019b; Cole et al. 2019; Chang et al. 2019; Azzi et al. 2019; van Hoek 2019; Schmidt and Wagner 2019; Chang et al. 2020; Yadav and Singh 2020; Gonczol et al. 2020; Öztürk and Yildizbaşi 2020; Sternberg et al.^2020^; Ghode et al. 2020a; Kamble et al. 2020; Nandi et al. 2020; Wang et al. 2020a, b, c; Yadav et al. 2020; De Giovanni 2020; Lohmer et al. 2020; Esmaeilian et al. 2020; Kopyto et al. 2020; Qian and Papadonikolaki 2020; Valle and Oliver 2020; Ada et al. 2021; Li et al. 2021a; Centobelli et al. 2021	Performance outcomes result in cost reduction (Nandi et al. 2020), as well as smart contracts, which reduce costs due to their ability to execute themselves (Min 2019)
Supply chain actors' trust improvement	Min 2019; Cole et al. 2019; Hald and Kinra 2019; Rejeb et al. 2019; Schmidt and Wagner 2019; Sheel and Nath 2019; Wang et al. 2019a; Gonczol et al. 2020; Bai and Sarkis 2020; Durach et al. 2020; Ghode et al. 2020a; Di Vaio and Varriale 2020; Liu and Li 2020; Tönnissen and Teuteberg 2020; Casino et al. 2020; Kayikci et al. 2020; Wang et al. 2020a, b, c; Köhler and Pizzol 2020; De Giovanni 2020; Lohmer et al. 2020; Duan et al. 2020; Fosso Wamba et al. 2020a; Shoaib et al. 2020; Xue et al. 2020; Agrawal et al. 2021; Liu et al. 2021; Rejeb et al. 2021; Qian and Papadonikolaki 2020; Ada et al. 2021; Al-Rakhami and Al-Mashari 2021; Centobelli et al. 2021; Rana et al. 2021; Sivula et al. 2021	BCT improves trust among networked partners in supply chain (Rejeb et al. 2019) and creates an environment that does not require personal trust between the involved parties (Schmidt and Wagner 2019)
Data security	Kim and Laskowski 2018; Treiblmaier 2018; Mao et al. 2018; Leng et al. 2018; Juma et al. 2019; Wang et al. 2019a, b; Hald and Kinra 2019; Rejeb et al. 2019; Queiroz et al. 2019; Gurtu and Johny 2019; Cui et al. 2019; Bai and Sarkis 2020; Durach et al. 2020; Li et al. 2020; Howson 2020; Kamble et al. 2020; Nandi et al. 2020; Casino et al. 2020; Kayikci et al. 2020; Pournader et al. 2020; Fosso Wamba et al. 2020a; Kopyto et al. 2020; Dutta et al. 2020; Wan et al. 2020; Shoaib et al. 2020; Sunny et al. 2020; Li et al. 2021a; Musamih et al. 2021; Mukherjee et al. 2021; Wang et al. 2021	BCT provides a security guarantee mechanism (Leng et al. 2018) and data security for information sharing purposes (Wang et al. 2019a)
Data visibility	Perboli et al. 2018; Mao et al. 2018; Min 2019; Kamble et al. 2019; Montecchi et al. 2019; Wang et al. 2019a, b; Cole et al. 2019; Queiroz et al. 2019; Hald and Kinra 2019; Azzi et al. 2019; van Hoek 2019; van Hoek 2020a; Chang et al.^2020^; Hackius and Petersen 2020; Durach et al. 2020; Rogerson and Parry 2020; Di Vaio and Varriale 2020; Tönnissen and Teuteberg 2020; Kayikci et al. 2020; Wang et al. 2020a, b, c; Yadav et al. 2020; Lohmer et al. 2020; Esmaeilian et al. 2020; Duan et al. 2020; Wan et al. 2020; Xue et al. 2020; Stranieri et al. 2021; Agrawal et al. 2021; Reddy et al. 2021	Data in BCT-based supply chain is visible to all stakeholders (Yadav et al. 2020), visibility increases because of real-time sharing data access in the network (Lohmer et al. 2020)
Product safety assurance	Perboli et al. 2018; Kshetri 2018; Juma et al. 2019; Kamble et al. 2019; Saberi et al. 2019; Montecchi et al. 2019; Cole et al. 2019; Azzi et al. 2019; Kamilaris et al. 2019; Mattke et al. 2019; Chang et al. 2020; Bai and Sarkis 2020; van Hoek 2020b; Durach et al. 2020; Rogerson and Parry 2020; Behnke and Janssen 2020; Kamble et al. 2020; Liu and Li 2020; Ghode et al. 2020b; Nandi et al. 2020; Casino et al. 2020; Fan et al. 2020; Kayikci et al. 2020; Köhler and Pizzol 2020; Duan et al. 2020; Kouhizadeh et al. 2021; Kramer et al. 2021; Agrawal et al. 2021; Li et al. 2021a; Yang et al. 2021	BCT enhances product safety by providing records of safety conditions (Cole et al. 2019), strengthens food safety assurance (Azzi et al. 2019), and helps to control more efficiently food-caused contaminations (Köhler and Pizzol 2020)
Efficiency improvement	Leng et al. 2018; Cole et al. 2019; Chang et al. 2019; Rejeb et al. 2019; van Hoek 2019; Cui et al. 2019; Gonczol et al. 2020; Durach et al. 2020; Sternberg et al. 2020; Choi 2020; Di Vaio and Varriale 2020; Tönnissen and Teuteberg 2020; Nandi et al. 2020; Köhler and Pizzol 2020; Esmaeilian et al. 2020; Duan et al. 2020; Fosso Wamba et al. 2020a; Kopyto et al. 2020; Dutta et al. 2020; Hastig and Sodhi 2020; Wan et al. 2020; Shoaib et al. 2020; Ada et al. 2021; Chen et al. 2021; Kayikci et al. 2021; Li et al. 2021a; Lim et al. 2021; Centobelli et al. 2021; Park and Li 2021; Rana et al. 2021	BCT boosts operational efficiency (Sternberg et al. 2020), as well as the overall efficiency of the system (Leng et al. 2018)
Information authenticity	Perboli et al. 2018; Mao et al. 2018; Leng et al. 2018; Juma et al. 2019; Saberi et al. 2019; Montecchi et al. 2019; Cole et al. 2019; O’Leary 2019; Azzi et al. 2019; Schmidt and Wagner 2019; Mattke et al. 2019; Cui et al. 2019; Cha et al. 2020; Durach et al. 2020; Li et al. 2020; Wong et al. 2020a; Choi 2020; Rogerson and Parry 2020; Di Vaio and Varriale 2020; Liu and Li 2020; Wong et al. 2020b; Ghode et al. 2020b; Nandi et al. 2020; Köhler and Pizzol 2020; Duan et al. 2020; Shoaib et al. 2020; Ahmad et al. 2021; Li et al. 2021a; Stranieri et al. 2021	BCT improves the information flow (Ghode et al. 2020b) and provides access to verified information and data (Perboli et al. 2018)
Intermediary elimination	Perboli et al. 2018; Montecchi et al. 2019; Wang et al. 2019b; Queiroz and Fosso Wamba 2019; Cole et al. 2019; Gurtu and Johny 2019; Hald and Kinra 2019; Gonczol et al. 2020; Durach et al. 2020; Öztürk and Yildizbaşi 2020; Li et al. 2020; Choi 2020; Howson 2020; Behnke and Janssen 2020; Casino et al. 2020; Yadav et al. 2020; De Giovanni 2020; Esmaeilian et al. 2020; Duan et al. 2020; Pournader et al. 2020; Dutta et al. 2020; Li et al. 2021b; Centobelli et al. 2021; Rana et al. 2021; Saurabh and Dey 2021	There is no need of a trusted authority when using BCT (Dutta et al. 2020) who would verify transaction attributes (Esmaeilian et al. 2020)
End consumers' trust improvement	Perboli et al. 2018; Fu and Zhu 2019; Kamble et al. 2019; Montecchiet al. 2019; Queiroz and Fosso Wamba 2019; Azzi et al. 2019; Kamilaris et al. 2019; van Hoek 2019; Gonczol et al. 2020; Bai and Sarkis 2020; Fan et al. 2020; Sternberg et al. 2020; Ghode et al. 2020a; Rogerson and Parry 2020; Casino et al. 2020; Köhler and Pizzol 2020; Fosso Wamba et al. 2020a; Shoaib et al. 2020; Xue et al.^2020^; Stranieri et al. 2021; Agrawal et al. 2021; Cao et al. 2021; Kayikci et al. 2021	BCT adoption in SC processes can boost the trust of a customer (Ghode et al. 2020a), consequently this results in extra sales and helps to find the best customer (Shoaib et al. 2020)
Transactions automation (smart contracts)	Cole et al. 2019; Hald and Kinra 2019; Chang et al. 2020; Kamilaris et al. 2019; Schmidt and Wagner 2019; Queiroz et al. 2019; Bai and Sarkis 2020; Durach et al. 2020; Li et al. 2020; Kamble et al. 2020; Tönnissen and Teuteberg 2020; Nandi et al. 2020; Wang et al. 2020a, b, c; Pournader et al. 2020; Kopyto et al. 2020; Shoaib et al. 2020;Bekrar et al. 2021;Büyüközkan et al. 2021; Li et al. 2021a; Lim et al. 2021; Lou et al. 2021; Rana et al. 2021	Smart contracts automatically execute payments once the contract terms and conditions are met (Wang et al. 2020a, b, c), enabling thus automatic trading of data between different SC network participants (Li et al. 2020)
"Single version of truth" access	Fu and Zhu 2019; Kamble et al. 2019; Cole et al. 2019; Rejeb et al. 2019; Chang et al. 2020; Azzi et al. 2019; Gurtu and Johny 2019; Yadav and Singh 2020; Hackius and Petersen 2020; van Hoek 2020b; Öztürk and Yildizbaşi 2020; Di Vaio and Varriale 2020; Ghode et al. 2020b; Nandi et al. 2020; Köhler and Pizzol 2020; Lohmer et al. 2020; Pournader et al. 2020; Fosso Wamba et al. 2020a, b; Kopyto et al. 2020; Wong et al. 2020b; Mukherjee et al. 2021	BCT retains permanent and tamperproof records of data and transactions (Schmidt and Wagner 2019), which guarantees a single truth access to various SC participants (Gonczol et al. 2020)
Supply chain speed improvement	Fu and Zhu 2019; Kamble et al. 2019; Cole et al. 2019; Rejeb et al. 2019; Chang et al. 2020; Azzi et al. 2019; Gurtu and Johny 2019; Yadav and Singh 2020; Hackius and Petersen 2020; van Hoek 2020b; Öztürk and Yildizbaşi 2020; Di Vaio and Varriale 2020; Ghode et al. 2020b; Nandi et al. 2020; Köhler and Pizzol 2020; Lohmer et al. 2020; Pournader et al. 2020; Fosso Wamba et al. 2020a; Kopyto et al. 2020; Ada et al. 2021; Li et al. 2021a	BCT use speeds up international shipment and documentation processing (van Hoek 2020b), as well as enables to respond to SC anomalies more rapidly (Rejeb et al. 2019)
Data immutability	Perboli et al. 2018; Kim and Laskowski 2018; Treiblmaier 2018; Min 2019; Juma et al. 2019; Queiroz and Fosso Wamba 2019; Hald and Kinra 2019; Chang et al. 2020; Kamilaris et al. 2019; Schmidt and Wagner 2019; Yadav and Singh 2020; Cha et al. 2020; Bai and Sarkis 2020; Behnke and Janssen 2020; Kamble et al. 2020; Fosso Wamba et al. 2020a; Dutta et al. 2020; Shoaib et al. 2020; Lou et al. 2021; Menon and Jain 2021; Rana et al. 2021	The nature of BCT transactional data is immutable (Behnke and Janssen 2020), meaning that data is resistant to any modification (Casino et al. 2019)
New value propositions	Perboli et al. 2018; Cole et al. 2019; Hald and Kinra 2019; Sheel and Nath 2019; Hackius and Petersen 2020; Sternberg et al. 2020; Ghode et al. 2020a; Howson 2020; Di Vaio and Varriale 2020; Ghode et al. 2020b; Queiroz et al. 2020; De Giovanni 2020; Esmaeilian et al. 2020; Fosso Wamba et al. 2020a; Dutta et al. 2020; Xue et al. 2020; Wang et al. 2020a, b, c; Karuppiah et al. 2021; Hong and Hales 2021; Stranieri et al. 2021; Sivula et al. 2021	BCT brings opportunities for new value propositions to the market (Hald and Kinra 2019) for instance in terms of facilitating conditions and social influence (Queiroz et al. 2020), including competitive advantage and better firm performance (Sheel and Nath 2019)
Transaction reliability	Mao et al. 2018; Leng et al. 2018; Saberi et al. 2019; Queiroz and Fosso Wamba 2019; Cole et al. 2019; Rejeb et al. 2019; Queiroz et al. 2019; Kamilaris et al. 2019; Schmidt and Wagner 2019; Mattke et al. 2019; Gonczol et al. 2020; Durach et al. 2020; Li et al. 2020; Wong et al. 2020a; Ghode et al. 2020b; Casino et al. 2020; Wang et al. 2020a, b, c; Duan et al. 2020; Fosso Wamba et al. 2020a	BCT is a reliable platform to collect data about transactions (Mao et al. 2018), which ensures fast and secure transactions without traditional authorities services (Casino et al. 2020)
Sustainability verification	Kshetri 2018; Saberi et al. 2019; Kamilaris et al. 2019; van Hoek 2019; Gurtu and Johny 2019; Gonczol et al. 2020; Bai and Sarkis 2020; Öztürk and Yildizbaşi 2020; Howson 2020; Rogerson and Parry 2020; Köhler and Pizzol 2020; Esmaeilian et al. 2020; Hastig and Sodhi 2020; Shoaib et al. 2020; Venkatesh et al. 2020; Wang et al. 2020a; Kouhizadeh et al. 2021; Centobelli et al. 2021	BCT links energy and water consumption information, which final consumer can then scan through barcode and see the environmental impact (van Hoek 2019), thus enabling a positive impact on environmentally and socially sustainable SCs (Rogerson and Parry 2020)
Fraud detection	Min 2019; Saberi et al. 2019; Azzi et al. 2019; Gurtu and Johny 2019; Gonczol et al. 2020; Öztürk and Yildizbaşi 2020; Rogerson and Parry 2020; Kamble et al. 2020; Wong et al. 2020a; Ghode et al. 2020b; Nandi et al. 2020; Köhler and Pizzol 2020; Fosso Wamba et al. 2020a; Wan et al. 2020; Balamurugan et al. 2021; Calvão and Archer 2021; Chen et al. 2021; Tezel et al. 2021	BCT adoption reduces fraudulent activities (Kamble et al. 2020), because any malicious attempt to change the information will be visible (Wan et al. 2020)
Risk reduction	Kshetri 2018; Min 2019; Fu and Zhu 2019; Montecchi et al. 2019; Hald and Kinra 2019; Gurtu and Johny 2019; Hackius and Petersen 2020; Choi 2020; Kamble et al. 2020; Kayikci et al. 2020; De Giovanni 2020; Duan et al. 2020; Dutta et al. 2020; Ada et al. 2021	BCT fully removes the risk in purchasing, services and transactions (De Giovanni 2020) and overall lowers the level of operational risk (Choi 2020)
Provenance assurance	Kim and Laskowski 2018; Montecchi et al. 2019; Gurtu and Johny 2019; Sternberg et al. 2020; Rogerson and Parry 2020; Kamble et al. 2020; Wang et al. 2020a, b, c; Dutta et al. 2020; Menon and Jain 2021; Musamih et al. 2021; Yiu 2021a; Kramer et al. 2021; Yiu 2021b	BCT's provenance feature allows SCs to make products more transparent (Gurtu and Johny 2019), enabling interorganizational provenance (Sternberg et al. 2020)
Counterfeit reduction	Perboli et al. 2018; Min 2019; Juma et al. 2019; Cole et al. 2019; Mattke et al. 2019; Cui et al. 2019; Gonczol et al. 2020; Rogerson and Parry 2020; Liu and Li 2020; Musamih et al. 2021; Yiu 2021a, b	BCT prevents the trade of fake or counterfeit assets (Min 2019) and protects SC from counterfeit products (Cui et al. 2019)
Supply chain collaboration improvement	Mao et al. 2018; Sternberg et al. 2020; Ghode et al. 2020b; Kayikci et al. 2020; Lohmer et al. 2020; Xue et al. 2020; Wang et al. 2020a, b, c; Hastig and Sodhi 2020; Musamih et al. 2021; Liu et al. 2021; Rejeb et al. 2021; Stranieri et al. 2021	BCT-based supply chains facilitate better collaboration among stakeholders (Mao et al. 2018), enhance trustful relationships and develop deeper cooperation (Xue et al. 2020)
Decentralized structure	Kshetri 2018; Leng et al. 2018; O’Leary 2019; Di Vaio and Varriale 2020; Kamble et al. 2020; Yadav et al. 2020; Duan et al. 2020; Fosso Wamba et al. 2020a; Kopyto et al. 2020; Wan et al. 2020; Falcone et al. 2021	BCT's decentralized structure (Kshetri 2018) can help eliminating information inequality (Duan et al. 2020)
Supply chain performance improvement	Hald and Kinra 2019; Chang et al. 2019; Sheel and Nath 2019; Ghode et al. 2020a; Choi 2020; Kayikci et al. 2020; Aslam et al. 2021; Kayikci et al. 2021; Li et al. 2021a; Centobelli et al. 2021	BCT distibuted system is expected to result in better performance throughout SC (Chang et al. 2019), getting rid off service fees can also potentially improve SC performance (Choi 2020)
Real-time auditing	Cole et al. 2019; Rejeb et al. 2019; Chang et al. 2020; Di Vaio and Varriale 2020; Kamble et al. 2020; Liu and Li 2020; Köhler and Pizzol 2020; Menon and Jain 2021; Rejeb et al. 2021	BCT-based supply chains enables audit of the transaction processes in time (Liu and Li 2020) and provides real time auditing via time-stamping (Cole et al. 2019)
Human error reduction	Perboli et al. 2018; Min 2019; Saberi et al. 2019; Wang et al. 2019b; Azzi et al. 2019; Bai and Sarkis 2020; Öztürk and Yildizbaşi 2020; Hastig and Sodhi 2020; Liu et al. 2021	BCT reduces the chances of human-caused errors (Perboli et al. 2018) that are hindering current operations (Öztürk and Yildizbaşi 2020)
Possible integration with other technologies	Rejeb et al. 2019; Gonczol et al. 2020; van Hoek 2020b; Kayikci et al. 2020; Duan et al. 2020; Pournader et al. 2020; Ada et al. 2021;Varriale et al. 2021	BCT pilot can be based on data feeds from other technologies (van Hoek 2020b), for instance combined with IoT devices (Duan et al. 2020)
Cyber security measures	Min 2019; Wang et al. 2019b; Gurtu and Johny 2019; Yadav and Singh 2020; Cha et al. 2020; Duan et al. 2020; Alkahtani et al. 2021	BCT-provided data security feature could protect SCs from cybercrimes and attacks (Wang et al. 2019b), such SCs are not feasible for data change or hacking (Yadav and Singh 2020)
Processes automation	Chang et al. 2019; Gurtu and Johny 2019; Öztürk and Yildizbaşi 2020; Tönnissen and Teuteberg 2020; Nandi et al. 2020; Kayikci et al. 2020; Tezel et al. 2021	Automated controls of processes are expected to disrupt existing SC ecosystem (Gurtu and Johny 2019) and increase the scope for SC process automation using smart contracts (Nandi et al. 2020)
Inventory reduction	Kamble et al. 2019; Cole et al. 2019; van Hoek 2019; Sternberg et al. 2020; Ada et al. 2021; Balamurugan et al. 2021; Kayikci et al. 2021	BCT adoption in SC is able to improve inventory management (Cole et al. 2019) and reduce inventory levels (Sternberg et al. 2020)
Paperwork reduction	Wang et al. 2019b; Azzi et al. 2019; Gonczol et al. 2020; van Hoek 2020b; Centobelli et al. 2021; Sundarakani et al. 2021	BCT reduces paperwork and administrative costs throughout SC (Azzi et al. 2019), as well as speeds up custom documentation for the shipment (van Hoek 2020b)
Bullwhip effect reduction	Perboli et al. 2018; Fu and Zhu 2019; Ghode et al. 2020a; Chen et al. 2021	The bullwhip effect can be minimized in BCT-based supply chains (Fu and Zhu 2019; Ghode et al. 2020a)
Planning optimization	Perboli et al. 2018; van Hoek 2020b; Esmaeilian et al. 2020; Büyüközkan et al. 2021	BCT-adopted supply chains support efficient operations planning (Esmaeilian et al. 2020), like shipment planning for instance, could begin earlier (van Hoek 2020b)
Vulnerabilities detection	Azzi et al. 2019; Köhler and Pizzol 2020; Stranieri et al. 2021	BCT gives insights into all the system’s vulnerabilities (Azzi et al. 2019) and discover firm weaknesses (Stranieri et al. 2021)
Forecast improvement	Perboli et al. 2018; Kamble et al. 2019	BCT helps the organizations to build and improve accurate demand forecasts (Perboli et al. 2018; Kamble et al. 2019)

**Table 3:** Disadvantages of blockchain technology in supply chain management context

**Disadvantages of BCT in SCM context**	**Authors**	**Explanation**
Information privacy concerns	Perboli et al. 2018; Kamble et al. 2019; Montecchi et al. 2019; Wang et al. 2019a; Hald and Kinra 2019; Chang et al. 2019; Schmidt and Wagner 2019; van Hoek 2019; Wang et al. 2019b; Gonczol et al. 2020; Öztürk and Yildizbaşi 2020; Sternberg et al. 2020; Ghode et al. 2020a; Rogerson and Parry 2020; Behnke and Janssen 2020; Liu and Li 2020; Ghode et al. 2020b; Yadav et al. 2020; Esmaeilian et al. 2020; Kopyto et al. 2020; Fosso Wamba et al. 2020b; Dutta et al. 2020; Wan et al.^2020^; Kouhizadeh et al. 2021; Agrawal et al. 2021; Li et al. 2021a; Centobelli et al. 2021; Xu et al. 2021; Yiu 2021a	On the interorganizational level, some organisations may assume open-access information as a competitive advantage, that is unwilling to be shared across SC (Kouhizadeh et al. 2021), thus information security is considered by practitioners as a potentil barrier for the BCT implementation in SC (Kopyto et al. 2020)
Scalability	Perboli et al. 2018; Mao et al. 2018; Min 2019; Juma et al. 2019; Chang et al. 2020; Azzi et al. 2019; Schmidt and Wagner 2019; van Hoek 2019; Gonczol et al. 2020; Cha et al. 2020; Öztürk and Yildizbaşi 2020; Li et al. 2020; Rogerson and Parry 2020; Behnke and Janssen 2020; Casino et al. 2020; Köhler and Pizzol 2020; Yadav et al. 2020; De Giovanni 2020; Esmaeilian et al. 2020; Duan et al. 2020; Pournader et al. 2020; Dutta et al. 2020; Chen et al. 2021; Musamih et al. 2021; Reddy et al. 2021; Yiu 2021a	Each node on the BCT network needs to store the entire history of transactions, so the growing size then becomes an issue ( Mao et al. 2018), and there is a limited capacity in handling a large amount of data (Azzi et al. 2019)
Low awareness and knowledge of technology	Treiblmaier 2018; Min 2019; Kamble et al. 2019; Wang et al. 2019a; van Hoek 2019; Montecchi et al. 2019; van Hoek 2020a; Chang et al. 2020; Gonczol et al. 2020; Hackius and Petersen 2020; Bai and Sarkis 2020; Fan et al. 2020; Öztürk and Yildizbaşi 2020; Wong et al. 2020b; Rogerson and Parry 2020; Köhler and Pizzol 2020; Duan et al. 2020; Fosso Wamba et al. 2020a; Wan et al. 2020; Büyüközkan et al. 2021; Chen et al. 2021; Stranieri et al. 2021; Katsikouli et al. 2021; Karuppiah et al. 2021; Li et al. 2021b; Reddy et al. 2021	BCT still does not represent a single monolithic artifact and is constantly being developed (Treiblmaier 2018), so there is still skepticism among practitioners due to low awareness about technology (Wong et al. 2020b)
High implementation cost	Perboli et al. 2018; Kamble et al. 2019; Cole et al. 2019; Schmidt and Wagner 2019; Wang et al. 2019a; Öztürk and Yildizbaşi 2020; Sternberg et al. 2020; Ghode et al. 2020a; Choi 2020; Howson 2020; Rogerson and Parry 2020; Wong et al. 2020a; Kayikci et al. 2020; De Giovanni 2020; Dutta et al. 2020; Wan et al. 2020; Chen et al. 2021; Musamih et al. 2021; Kshetri 2021; Li et al. 2021a; Lim et al. 2021; Stranieri et al. 2021	BCT platforms is expensive, especially for small-scale companies (Howson 2020) so the adoption of it may not pay off (Cole et al. 2019)
Low quality data input	Wang et al. 2019b; Chang et al. 2019; Rejeb et al. 2019; Azzi et al. 2019; Schmidt and Wagner 2019; Cha et al. 2020; Sternberg et al. 2020; Ghode et al. 2020a; Howson 2020; Rogerson and Parry 2020; Behnke and Janssen 2020; Ghode et al. 2020b; Köhler and Pizzol 2020; Esmaeilian et al. 2020; Duan et al. 2020; Pournader et al. 2020; Kopyto et al. 2020; Dutta et al. 2020; Fosso Wamba et al. 2020b; Chen et al. 2021; Stranieri et al. 2021	Information stored in BCT network is only as accurate as the incoming raw data (Wang et al. 2019b), since data cannot be further changed after it entered, there is a chance of a poor quality data (Köhler and Pizzol 2020)
Data manipulation / security concerns	Kamilaris et al. 2019; Cui et al. 2019; Behnke and Janssen 2020; Liu and Li 2020; Ghode et al. 2020b; Yadav et al. 2020; Esmaeilian et al. 2020; Wang et al. 2019a; Kopyto et al. 2020; Musamih et al. 2021; Yiu 2021a; Agrawal et al. 2021; Karuppiah et al. 2021; Yiu 2021b	Security problem is emergent for open access BCT networks (Liu and Li 2020), even though it offers advanced security, there are still high risks of funds losses (Kamilaris et al. 2019)
Lack of physical traceability	Kamilaris et al. 2019; Chang et al. 2020; Yadav and Singh 2020; Howson 2020; Köhler and Pizzol 2020; Esmaeilian et al. 2020; Kopyto et al. 2020; Rao et al. 2021; Stranieri et al. 2021; Yiu 2021b	There is no certainty if the link between digital records and physical goods is verdically established (Esmaeilian et al. 2020), thus it makes confusion about real quality conditions of physical products (Stranieri et al. 2021)
High energy consumption	Öztürk and Yildizbaşi 2020; Kamble et al. 2020; Yadav et al. 2020; Esmaeilian et al. 2020; Kopyto et al. 2020; Musamih et al. 2021; Lim et al. 2021; Kramer et al. 2021	BCT consumes excessive energy (Öztürk and Yildizbaşi 2020) due to high number of network participants (Kopyto et al. 2020)
Lack of Blockchain suitability in some product/context	van Hoek 2020a; van Hoek 2020b; Ghode et al. 2020b; De Giovanni 2020; Shoaib et al. 2020; Kshetri 2021; Menon and Jain 2021	Low cost products may not have a need in traceability if value of it is not justified for such high-cost technology (van Hoek 2020a), so it might not be a right technology for any SC context (van Hoek 2020b)
Lack of central authority / coordination – 6	Wang et al. 2019b; Kamilaris et al. 2019; Cha et al. 2020; Fan et al. 2020; Wang et al. 2020a, b, c; Karuppiah et al. 2021	Lack of central authority might be missing for resolving potential disputes (Wang et al. 2019b), especially for private chains (Cha et al. 2020)
Difficulties in smart contract adoption	Cole et al. 2019; Hald and Kinra 2019; Esmaeilian et al. 2020; Kopyto et al. 2020; Menon and Jain 2021	Poor smart contact coding may lead to problems (Cole et al. 2019) and difficulties in their further modification (Hald and Kinra 2019)
ROI concerns	Wang et al. 2019b; Cole et al. 2019; van Hoek 2019; van Hoek 2020a	The progress of such a disruptive technology as BCT might be slow due to low level of knowledge, thus resulting in a lack of visible financial benefits (Wang et al. 2019b). The costs and the ROI of BCT implementation to SC are still obscure (van Hoek 2019)

**Table 4:** Constraints of blockchain technology in supply chain management context

**Constraints of BCT in SCM context**	**Authors**	**Explanation**
Early stage of Blockchain adoption in practice	Fu and Zhu 2019; Saberi et al. 2019; Wang et al. 2019b; van Hoek 2020a; Schmidt and Wagner 2019; Gonczol et al. 2020; Hackius and Petersen 2020; Bai and Sarkis 2020; van Hoek 2020b; Öztürk and Yildizbaşi 2020; Ghode et al. 2020a; Wong et al. 2020b; Kamble et al. 2020; Fosso Wamba and Queiroz 2020b; Kayikci et al. 2020; Köhler and Pizzol 2020; Fosso Wamba et al. 2020b; Xue et al. 2020; Büyüközkan et al. 2021; Chen et al. 2021; Collart and Canales 2021; Liu et al. 2021; Reddy et al. 2021; Teodorescu and Korchagina 2021	BCT is still at the stage of testing and analysis, and does not yet have examples of large-scale application in practice (Fu and Zhu 2019), therefore implemention in such an early stage brings potential risks for organizations (Schmidt and Wagner 2019)
Lack of organizational policies/legal regulations	Min 2019; Saberi et al. 2019; Chang et al. 2019; Chang et al. 2020; Gonczol et al. 2020; Hackius and Petersen 2020; Howson 2020; Rogerson and Parry 2020; Kamble et al. 2020; Ghode et al. 2020b; Fosso Wamba and Queiroz 2020b; Kayikci et al. 2020; Yadav et al. 2020; Esmaeilian et al. 2020; Wang et al. 2019a; Duan et al. 2020; Fosso Wamba et al. 2020a; Dutta et al. 2020; Musamih et al. 2021; Katsikouli et al. 2021; Wang et al. 2020a, b, c; Chen et al. 2021; Karuppiah et al. 2021; Li et al. 2021a	Lack of standards, legal issues and protocols (Chang et al. 2019) poses a problem in BCT recognition and its features in laws and regulations (Hackius and Petersen 2020)
Technology complexity	Min 2019; Kamble et al. 2019; Wang et al. 2019a; Wang et al. 2019b; Azzi et al. 2019; Hoek 2019; Gonczol et al. 2020; Öztürk and Yildizbaşi 2020; Sternberg et al. 2020; Wong et al. 2020a; Behnke and Janssen 2020; Kamble et al. 2020; Fosso Wamba and Queiroz 2020a; Wan et al. 2020; Tönnissen and Teuteberg 2020; Köhler and Pizzol 2020; Esmaeilian et al. 2020; Fosso Wamba et al. 2020a; Sunny et al. 2020; Wong et al. 2020b; Li et al. 2021a; Mathivathanan et al. 2021; Stranieri et al. 2021	Practitioners have a need in developing greater understanding of how to integrate BC into their SC processes (Hoek 2019), so this challenge is technical and native by its nature due to the current state of the BCT (Gonczol et al. 2020)
Supply chain participants’ inertia	Perboli et al. 2018; Kshetri 2018; Juma et al. 2019; Montecchi et al. 2019; van Hoek 2019; Gonczol et al. 2020; Sternberg et al. 2020; Rogerson and Parry 2020; Behnke and Janssen 2020; Di Vaio and Varriale 2020; Liu and Li 2020; Köhler and Pizzol 2020; Wang et al. 2019a; Duan et al. 2020; Fosso Wamba et al. 2020a; Wan et al. 2020; Kouhizadeh et al. 2021; Caldarelli et al. 2021; Kayikci et al. 2021; Sharma et al. 2021;Valle and Oliver 2020; Yang et al. 2021; Yiu 2021a	There could potentially arise an unwillingness to share valueable information across SC (Wang et al. 2019a) and without all SC entities participation the disclosure of BCT's fullpotential in SCM will not be possible (Kshetri 2018)
Interoperability	Min 2019; Rejeb et al. 2019; O’Leary 2019; van Hoek 2020b; Öztürk and Yildizbaşi 2020; Howson 2020; Liu and Li 2020; Wong et al. 2020a; Kayikci et al. 2020; Wang et al. 2020a, b, c; Yadav et al. 2020; De Giovanni 2020; Dutta et al. 2020; Menon and Jain 2021; Musamih et al. 2021	It is still unclear how BCT and its systems will interoperate and integrate with other technologies (Rejeb et al. 2019), so the interface between BCT and existing enterprise systems is a very important issue (Liu and Li 2020)
Information reliability assurance	Montecchi et al. 2019; Chang et al. 2019; Azzi et al. 2019; Cui et al. 2019; Cha et al. 2020; Sternberg et al. 2020; Ghode et al. 2020a; Rogerson and Parry 2020; Kayikci et al. 2020; Esmaeilian et al. 2020; Duan et al. 2020; Kopytoet al. 2020; Fosso Wamba et al. 2020b	SC network participants need to make sure that data input is safe and reliable before storing it in an immutable ledger (Azzi et al. 2019), because historical data needs to be reliable, non-tempered, but also balancing between transparency and confidentiality issues (Ghode et al. 2020a)
Lack of infrastructure for Blockchain adoption	Kamble et al. 2019; Öztürk and Yildizbaşi 2020; Wong et al. 2020a; Behnke and Janssen 2020; Di Vaio and Varriale 2020; Wong et al. 2020b; Yadav et al. 2020; Queiroz et al. 2019; Kouhizadeh et al. 2021, Sivula et al. 2021	BCT sill lacks an organized ecosystem for starting its application to SC (Kamble et al. 2019), including the inadequacy of current existing technological infrastructure (Öztürk and Yildizbaşi 2020)
Lack of trust between supply chain actors	Chang et al. 2020; Sternberg et al. 2020; Wong et al. 2020b; Behnke and Janssen 2020; Liu and Li 2020; Ghode et al. 2020b; Yadav et al. 2020; Kopyto et al. 2020	The fact that actors may have different relationships with each other in different situations is complicating BCT adoption to SCs, e.g. the same entities can be competitors in the one market segment and customer–supplier in other (Behnke and Janssen 2020). Thus, trust issues among SC participants become a crucial issue to overcome in order to implement the system (Liu and Li 2020)
Cultural differences of supply chain participants	Kshetri 2018; Öztürk and Yildizbaşi 2020; Liu and Li 2020; Wong et al. 2020a; Ghode et al. 2020b; Queiroz et al. 2020; Fosso Wamba et al. 2020a; Kayikci et al. 2021	Some SC participants may be located in developing or least developed countries, which obstructs their participation in BCT adoption (Kshetri 2018). Moreover, organizational culture may result in a different decision-making process when adopting BCT to SC (Wong et al. 2020a)
Standardization of processes	van Hoek 2019; Gonczol et al. 2020; Behnke and Janssen 2020; Hastig and Sodhi 2020; Sunny et al. 2020; Chen et al. 2021	The key boundary conditions is the standardization of traceability processes, quality compliance etc. among all SC parties involved within a given network (Gonczol et al. 2020), so standardization towards BCT platform adoption is required (Behnke and Janssen 2020)
Limited technology access	Saberi et al. 2019; Kamilaris et al. 2019; Howson 2020; Sunny et al. 2020; Wu et al. 2021; Vivaldini and de Sousa 2021	While BCT is able to connect complex global SCs, the information infrastructure required to operate might prevent access to markets for new users ( Kamilaris et al. 2019), so technology access limitation is also an important issue to overcome in future (Saberi et al. 2019)

The three most reported advantages of BCT use in the SCM context (Table 2) are (1) transparency and traceability, (2) cost reduction, and (3) supply chain actors’ trust improvement. This can be explained by the nature of the decentralized distributed ledger, that creates a trusted environment among SC players (Büyüközkan et al. 2021) and has beneficial outcomes in terms of enhancing the efficiency of SCs. The transparency afforded by BCT spreads to processes and products, thereby ensuring information authenticity (Bai and Sarkis 2020).

The three most mentioned disadvantages of BCT use in the SCM context (Table 3) are (1) information privacy concerns, (2) scalability, and (3) low awareness and knowledge of technology. Overall, these three disadvantages can be explained by the fact that this technology is still novel, and thus doubts and concerns remain regarding its application in the SCM context (Fosso Wamba et al. 2020b). Moreover, low awareness may arise not only from the side of SC partners, but also from the side of final consumers’ low acquaintance with BCT features; consequently, this might be a critical factor of adoption renouncement (Fan et al. 2020).

Further, the three most mentioned constraints of BCT use in the SCM context (Table 4) are (1) the early stage of blockchain adoption in SCM practices, (2) lack of organizational policies/legal regulations, and (3) technology complexity. Overall, these constraints highlight the novelty of BCT and practical considerations in its application. Early-stage creating barriers in terms of implementation indicates that future research must be conducted to overcome them (Saberi et al. 2019).

Thus, there is a wide range of features reported in the literature related to the implementation of BCT in SCM. Their nature and areas of impact vary, and systematization is required to perceive the full extent of their interrelations. Consequently, these areas of impact are further analyzed to highlight the interrelated influence of the perceived advantages, disadvantages, and constraints.

The features of BCT can be considered from different perspectives, and as shown in Tables 2, 3 and 4, they are extremely varied and somewhat contradictory. Inspired by a study found in the extant literature, where BCT adoption was explored from the point of view of Malaysian small-medium enterprises (SMEs) under three dimensions: technological, organizational, and environmental (Wong et al. 2020a), we wanted to bring a general view on the adoption process and which benefits and challenges BCT can cause to SCs. Thus, we propose an attempt to group and systematize BCT features into the comprehensive dimensions of the impact of technology adoption under general SCM context, not attached to a specific context/sample group, in order to see in which areas and in what ways it can influence SC improvements and performances.

Novel digital technologies play a major role in managing information flow and processes for operations and SCs (Wong et al. 2020a), both scholars and practitioners are aiming to improve operations and processes of business practices with capabilities that disruptive technologies bring (Boutkhoum et al. 2021). When examining BCT from the SC perspective, its application in large-scale production is justified (Fu and Zhu 2019); it acts as a unified ledger for manufacturing and processes (Cole et al. 2019). Thus, it improves SC processes because there is no need for an intermediary (Öztürk and Yildizbaşi 2020). As a result, it optimizes and enhances operations and inbound efficiency (Perboli et al. 2018). Hence, the first dimension of impact is characterized as organic SCM activities; because it mostly relates to process efficiency and operations in the SCM context, the first dimension of impact is labeled as ‘operations and processes’.

The main component of any SC is its entities/participants. BCT connects multiple stakeholders (Mao et al. 2018), reducing trust issues (Kamble et al. 2019) and potentially removing third-party actors with more power over prices and processes who can become manipulative (Wang et al. 2019a, b). Thus, BCT plays a role in collaborative behavior among stakeholders (Chang et al. 2019), improves the information flow between partners (Hackius and Petersen 2020), and provides authenticity and knowledge provenance to end consumers (Montecchi et al. 2019). As a disruptive technology, implementation of BCT requires commitment from upper management as well, to encourage team spirit in the adoption process and to help overcome related challenges (Zhou et al. 2020). It was recently revealed that social influence, or in the SC context a partnership experience, plays a substantial role when introducing novel systems (Alazab et al. 2021). Consequently, the second dimension of impact is aimed to shed light on those BCT features that impact relationships among organizations’ representatives, and is called ‘supply chain relationships’.

Considered as the ‘latest transformative innovation’ (Schmidt and Wagner 2019), characterized by innate features such as its immutability and distributed nature (Gonczol et al. 2020), BCT requires SC participants to be open-minded to innovative ideas (Min 2019). The inherent characteristics of BCT can enhance and optimize SC potential (Juma et al. 2019) while building an authentic and trustworthy ecosystem (Azzi et al. 2019) with a novel view on data access. One of the success factors for a blockchain-based SC is ‘system strength’, which encompasses the representative capabilities of BCT, including a trustless environment and network resistance to attacks (Shoaib et al. 2020). The need to examine innovation trends of BCT-based solutions for business networks was highlighted (Dehghani et al. 2020), expected to bring innovation in both aspects: technological and managerial (Dehghani et al. 2021). Overall, this highlights the need in the third dimension of impact, that would represent impact of the distinctive features of BCT itself: named as ‘innovation and data access’ dimension.

Above-mentioned dimensions are rather emerging naturally when addressing the impact of BCT in SCM practices, thus further translated into the dimensions, it brings clarity for the classification of specific BCT features. The logic behind defining these dimensions of impact is to represent them graphically as clusters and classify the characteristics from Tables 2, 3 and 4 within the formulated dimensions to determine the existence of interrelationships and the overlapping areas that arise between dimensions.

### Advantages of blockchain technology when implemented in supply chains

**Fig. 2:**
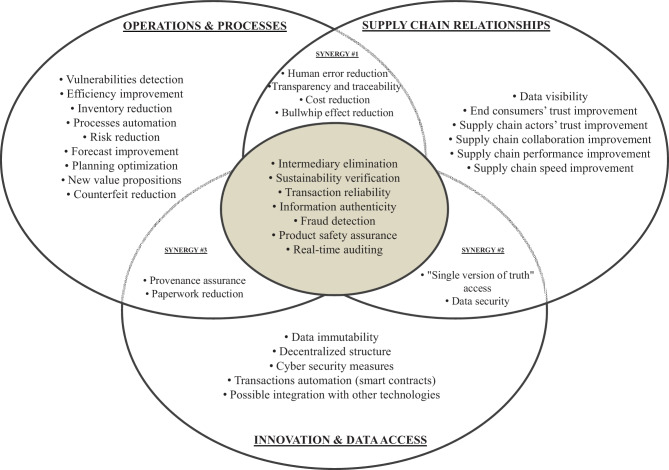
Venn diagram for advantages of blockchain technology implementation in supply chain management

Figure 2 represents the advantages classified within each of the three impact dimensions. For graphical representation purposes, a Venn diagram highlights the coexistence and overlap within these dimensions. It is relevant to understand, that overlapping areas represent features intrinsic to a mix of different dimensions; the overlapping areas are from now on going to be called “synergies”.

Synergy #1, between ‘operations and processes’ and ‘supply chain relationships’, indicates advantages such as the reduction of human error, which is enabled by smart contracts features, that are characterized by self-verification and self-integrity, allowing elimination of previously trusted third parties (Deebak and Fadi 2021). It therefore potentially results in reduced errors in order fulfilment (Min 2019), process deployment (Saberi et al. 2019), and reduced paperwork errors (Wang et al. 2019a, b). Decentralized BCT-based networks also reduce risk in operations (Öztürk and Yildizbaşi 2020) in data manipulation coming from the administrative side in current centralized systems (Hewa et al. 2021). Cost reduction stems as well from the automation of transactions (Cole et al. 2019): automation simplifies SCs because intermediaries are no longer needed (Montecchi et al. 2019), which substantially reduces transaction costs (Yadav et al. 2020).

Synergy #2 relates to features that bring advantages from the perspectives of ‘supply chain relationships’ and ‘innovation and data access’ of BCT. The advantages are clearly discernible; BCT, as a digital transaction ledger (Kamilaris et al. 2019), can store everything within the SC’s memory, and any actor can access it at any time (Hald and Kinra 2019), thus enabling ‘timely information acquisition and sharing’(Wong et al. 2020b, p.2102). BCT also provides highly secure access to data (Kim and Laskowski 2018); thus, its immutable nature combined with cryptographic security leads to a new level of trust between actors (Treiblmaier 2018).

Synergy #3, which lies between ‘operations and processes’ and ‘innovation and data access’, is extremely interesting. The BCT-based flow of information reduces physical risks to individuals, preventing potential health harm to consumers, therefore protecting entities from failures through the availability of transparent records; it increases the knowledge provenance of a product (Montecchi et al. 2019), both to SC actors and end customers (Rogerson and Parry 2020). For instance, shipping processes reduce trade documentation (Wang et al. 2019a, b), enable customs documentation processing to commence earlier (van Hoek 2020b), and decrease paperwork in general (Gonczol et al. 2020).

This classification allows us to identify the key advantages of all three dimensions of impact. This centre area in the diagram cannot be considered to represent the ‘main’ advantages, but it does reveal the characteristics that are common to all three dimensions:

1. *Intermediary elimination:* There is no need to verify transactions through a third party (Pournader et al. 2020) or a trusted authority (Dutta et al. 2020).
2. *Sustainability verification:* BCT enables the visibility of water and energy consumption throughout the SC processes (van Hoek 2019), as, when applied to a fast-fashion industry (Wang et al. 2020a), thus helping achieve emergent sustainability goals (Bai and Sarkis 2020). BCT can also shed more light on social sustainability: it can bring transparency into working safety and conditions, preventing working exploitation practices and inequality (Venkatesh et al. 2020).
3. *Transaction reliability:* BCT assures transaction authenticity (Leng et al. 2018); being fully traceable, organisations can see the origins of transactions (Queiroz and Fosso Wamba 2019).
4. *Information authenticity:* This implies both the availability of verified information accessible to any entity part of the network (Nandi et al. 2020) and backward monitoring available to the end consumer (Casino et al. 2019).
5. *Fraud detection:* This refers to entity verification, given that the authenticity of each device is verified (Cui et al. 2019), which guarantees that each user is authorised (Duan et al. 2020) and enables the detection of potentially fraudulent activities within the network (Kshetri 2018).
6. *Product safety assurance:* This mainly emerged among food SCs, where the characteristics of food, its storage conditions, etc., can improve safety and avoid contamination (Kamilaris et al. 2019) by enabling the on time recall of products (Azzi et al. 2019). Safety assurance is also applicable for other types of SCs; BCT ensures the safety and quality of products for end consumers (Montecchi et al. 2019);
7. *Real-time auditing:* Owing to its decentralised nature (O’Leary 2019) and the automation of processes, BCT enables real-time auditing through time-stamping (Cole et al. 2019).

### Disadvantages of blockchain technology when implemented in supply chains

**Fig. 3:**
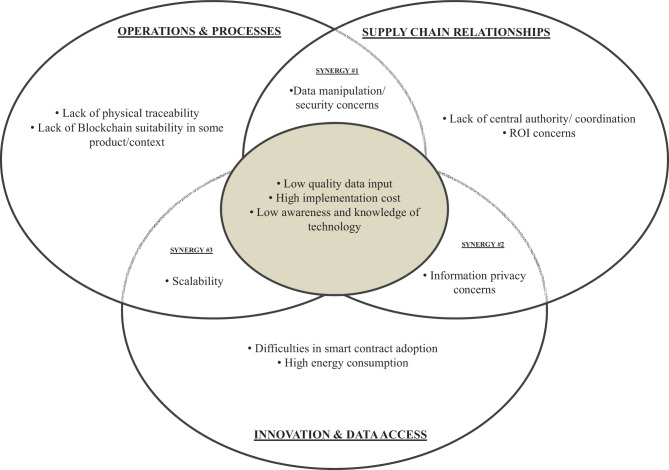
Venn diagram for disadvantages of blockchain technology implementation in supply chain management

Interestingly, the three dimensions of impact have some overlap in terms of advantages, disadvantages, and constraints. Figure 3 provides an illustrative example of how the same feature can be read an advantage or disadvantage, depending on the context.

At Synergy #1, between ‘operations and processes’ and ‘supply chain relationships’, security concerns and the data manipulation are noted as disadvantages. Data manipulation is mainly a characteristic of open access blockchain networks (Liu and Li 2020). It is demonstrated by examples of hacking attack incidents with some cryptocurrencies (Wang et al. 2019a), where a data miner could invade at one point of a BCT network to compromise original nodes to get a fraudulent high revenue (Karuppiah et al. 2021). Even in private blockchain networks, this is a potential issue in cases where, for example, the account owner accidentally loses the pair of private keys, thus losing access to the network and authorisation (Kamilaris et al. 2019), and in the case of such security breach, documents can potentially be tampered, resulting in disturbance of SC operations. However, the order placed in the network cannot be tampered (Ada et al. 2021); thus, even if BCT leaves opportunities for data manipulation or infringement, when compared with a centralized network, peer-to-peer BCT architecture it is still a more secure system (Büyüközkan et al. 2021).

Synergy #2, between ‘supply chain relationships’ and ‘innovation and data access’, is a similar, albeit slightly different, disadvantage: information privacy concerns. Such concern is mainly explained by the unwillingness of stakeholders to share full information along the complex multi-tier SCs (Perboli et al. 2018). The perspective on BCT-enabled transparency is controversial upon different tiers of players, e.g. upper tiers have more motivation to keep its information confidential from lower tiers of SC (Öztürk and Yildizbaşi 2020). Thus, it is crucial for SC players to jointly define to what extent of detailed information network should go, in order to keep information transparency at justifiable level, and to prevent misuse of information (Behnke and Janssen 2020). This issue may as well occur when a stakeholder considers valuable information a competitive advantage and is unwilling to share it with other participants in the SC (Kouhizadeh et al. 2021).

Finally, Synergy #3, between ‘innovation and data access’ and ‘operations and processes’, relates to scalability issues, which depend on the novelty of the technology itself and the efficiency of operations. The scalability issue is explained by the fact that each blockchain node needs to contain the network’s entire history (Mao et al. 2018), which limits the capacity to deal with big data and limits data storage (Cha et al. 2020).

One of the disadvantages in the centre, overlapping area relates to the high cost of implementing this technology, which seems to contradict a feature shown in Fig. 2: that BCT can reduce costs due to its innate qualities. Both of these arguments arise when an organisation considers implementing BCT- it has a high investment cost (Öztürk and Yildizbaşi 2020), and such platforms can be too expensive for small-scale stakeholders within the chain (Howson 2020). The other two central issues can be seen as a continuation of each other: low-quality data input means possible errors related to the incoming data to the platform (Stranieri et al. 2021), due to a lack of sufficient knowledge about technology and the concepts behind it (Öztürk and Yildizbaşi 2020), which are constantly being developed (Treiblmaier 2018).

### Constraints of blockchain technology when implemented in supply chains

**Fig. 4:**
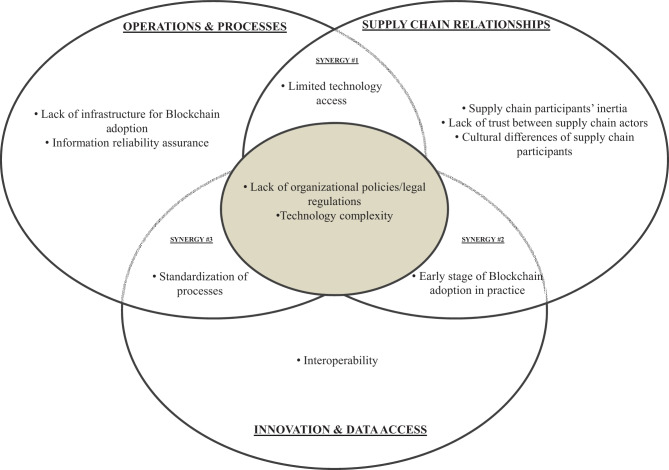
Venn diagram for constraints of blockchain technology implementation in supply chain management

When assessing the constraints of BCT in the SC context, most constraints turned out to be organisation- and technology-related issues in terms of practice, which are shown in Fig. 4.

For example, Synergy #1 reveals limitations in accessing the technology (Saberi et al. 2019). Although BCT can connect complex international SCs, it can hinder or prevent new players and stakeholders from joining the system (Kamilaris et al. 2019).

Synergy #2 reveals another typical constraint for practitioners in the early stages of BCT adoption. While still in the testing and analysis stages (Fu and Zhu 2019), there is a limited understanding of how to adopt and roll it out effectively (van Hoek 2019), which creates uncertainty in the decision-making process (Ghode et al. 2020b), thereby creating organisational risk (Schmidt and Wagner 2019).

The standardisation of processes in Synergy#3 is also related to the early stages of technology adoption. It primarily concerns uncertainty regarding BCT integration into SC processes (van Hoek 2019), encompassing traceability, compliance, and quality requirements (Gonczol et al. 2020).

The constraint issues in the centre area regarding BCT implementation in the SC context may require considerable time to overcome because they are common to all three dimensions. There is still a lack of government regulatory processes (Min 2019), as well as a lack of legal standards and protocols (Chang et al. 2019), highlighting the need to develop new BCT-related governing policies (Ghode et al. 2020a). Another common difficulty is the complexity of the BCT. Non-technical experts lack sufficient knowledge (Gonczol et al. 2020) regarding the numerous new concepts that practitioners need to be aware of, such as smart contracts, cryptography, programming languages, and much more (Köhler and Pizzol 2020). Technical knowledge, therefore, needs to be addressed in the future (Kamble et al. 2019).

## Discussion

There were three aims of this study: (1) to explore the dimensions of the impact of BCT implementation in SCM; (2) to explore the synergies of these dimensions; and (3) to explore the virtuous and vicious cycles of the adoption of BCT use in SCM. The study’s findings reveal the most emergent and distinctive features of BCT in the SCM context, enabling us to visualise overlaps and interrelationships between the dimensions of impact schematically. The remainder of this section identifies the theoretical contributions, managerial implications, and areas for future research.

### Theoretical contribution

The theoretical contribution of this study is twofold. First, our study proposes a systematisation of BCT features in the SCM context that have been analysed, formulated, and classified in a unified manner, in contrast to the wide dispersion of classifications in prior literature. One of the reviewed articles adopted a similar approach: an SLR where potential benefits and challenges related to BCT were identified and later generalised and grouped into ‘high-value benefits/challenges’ (Fosso Wamba et al. 2020a, b). The proposed systematisation in the present study contributes to an understanding of the features of BCT in the SCM context that is up-to-date and summarised in Tables 2, 3 and 4. This is important to fully comprehend the breadth of BCT features and their meaning in the SCM context.

In addition to the aforementioned study, other prior studies have adopted a similar approach, including listing the critical success factors for BCT adoption in SCs (Yadav and Singh 2020), categorising performance-measure features (Bai and Sarkis 2020), focusing on benefits and barriers (Kamilaris et al. 2019), and summarising the advantages and challenges of BCT adoption (Schmidt and Wagner 2019; Gonczol et al. 2020). However, the contribution of the present study goes beyond prior studies to provide a generalised vision and holistic understanding of the existing features of BCT in the SCM context, without limitation to a specific industry or area of application.

The second theoretical contribution of this research is the identification of the three dimensions of the impact of BCT adoption in SCM. The ability to distinguish BCT features between the three dimensions and their intersections (Figs. 2, 3 and 4) alone brings a fresh perspective to this area of study. Thus, it improves our understanding of considerations under the detected dimensions of impact and their synergies, systematises knowledge of BCT in the SCM context, and broadens the perspective to a more holistic approach. To the best of our knowledge, these operations management dimensions have not yet been considered in the context of BCT features in SCM. Similar contributions have been made, but only in grouping the success factors of BCT-based SCs (Shoaib et al. 2020). Therefore, the present study advances the literature on BCT adoption in SCM, revealing new perspectives on BCT features’ consideration and grouping.

### Managerial implications

The findings of this research clarify the BCT features for SCM practitioners. Firms intending to begin a pilot for BCT adoption in their SC processes are now able to, by examining the proposed diagrams (Figs. 2, 3 and 4), critically assess, for instance, whether the advantages of BCT adoption outweigh the disadvantages and constraints in their specific SC context. Thus, SC managers will also be able to assess the consequences of their investment in one of the dimensions of impact, noting the existence of an impact on the other two dimensions, given that all three dimensions are interrelated and overlapping. Practitioners can position their SCs to benefit from the positive synergetic effect from BCT advantages while decreasing the counter-synergetic effect. Another implication is that practitioners should consider the constraints, which would allow them to, for example, apply the ‘Solution Canvas’ for BCT adoption based on their unique SCs, as done in previous research (Perboli et al. 2018).

Prior literature has highlighted the benefits and challenges of BCT adoption only in the context of specific SCs (Dutta et al. 2020), which suggests another opportunity for practitioners. The identified features in Tables II–IV could also be used and applied to a specific industrial area of interest in order to build a practical understanding of the features in different areas of application. One of the reviewed articles coded performance categories, identifying the impact of BCT on them (Stranieri et al. 2021), which represents another opportunity for practitioners. The features identified in this study could be applied to specific performance categories to evaluate the need for BCT adoption. BCT is being discussed actively and ‘promises a revolutionary approach to data storage which could give rise to new business models’ (Wong et al. 2020b, p.2115). In this context, the present study provides a veridical picture of not only the virtuous but also the vicious cycles of adopting BCT in SCM, which is relevant for practitioners in terms of improving their understanding before beginning a pilot.

### Future research opportunities

The identification of dimensions of the impact of BCT in operations area and SCs in specific opens many doors for future research. One of the most interesting and valuable future paths would be to apply the identified BCT dimensions of impact to a specific SCM area. For instance, it could be applied to cross-border logistics (Chang et al. 2020), e-commerce SCs (Liu and Li 2020), construction industry (Tezel et al. 2021), automobile SCs (Ada et al. 2021) and other industries, to determine whether there are differences in dimensions between specific areas. It would be interesting to examine differences between the SCM areas themselves, and differences between industries to reveal the key motivation factors and constraints of BCT adoption in various industries.

Moreover, the interaction between dimensions and synergies can vary in the same industry for different types of products. As one of the most common areas of BCT applications are food SCs, it would be valuable to examine dimensions separately for different types of food, e.g. marine conservations (Howson 2020), perishable food products (Kayikci et al. 2021), agri-food (Menon and Jain 2021) and see if the type of product plays a role in the perception of dimensions and features of BCT. It would be as well important to see the perspectives on the dimensions and synergies from the view of multiple tier SC players (Khan et al. 2021), as depending on the SC tier, the motivation and reasons for BCT adoption may vary.

Also, future studies could examine whether dimensions of impact and various cycles for manufacturing cases are different from those for service providers. Another research avenue is measuring differences in the large-scale adoption of BCT to SCM between for-profit and non-profit organisations, such as governmental pilots and business pilots. Further, inspired by existing research (Nandi et al. 2020), a framework for implementing BCT in SCM could be built based on the detected features and dimensions of impact to develop an understanding of their outcomes concerning SCM. We also suggest verifying this study’s proposed dimensions of impact through empirical research, including, for example, separate groups of various SC players, grouped based on their supply chain position (e.g. upper tiers separately from the lower tiers SC) to gather unbiased empirical data based on the views of a particular SC position. Such comparison of views on BCT adoption from different SC players would enrich the area of research with an understanding of the motivation for potential BCT pilots from different perspectives. It would bring value to both scholars and practitioners.

## Conclusions and limitations

To the best of our knowledge, this study is the first attempt of defining and exploring the dimensions of the impact of BCT implementation in SCM context. Not only we classify features of BCT under the proposed dimensions, but the vicious and virtuous cycles of dimensions’ synergies were also explored and discussed. This study provides room for future researchers to empirically investigate the proposed dimensions and their impacts on real use-cases/ pilots of BCT in the SCM area.

Although this study reveals valuable findings, it has limitations. Our study is an SLR, relying on prior studies to extract our findings, and due to the novelty of the research area, it is only based on 139 chosen studies. For example, BCT features have been identified in case studies (van Hoek 2019), and the benefits and challenges of BCT application have been identified based on interviews with SC experts (Wang et al. 2019a, b). Our findings are thus limited by the prior studies reviewed, including the methodologies used in prior studies. Therefore, it is crucial to further explore the field based on empirically collected data, using various data collection techniques, not limiting the study to be quantitative or qualitative.

We hope that this study will help create a fresh perspective on the dimensions of BCT adoption in the SC context and reveal new opportunities for managers and firms to understand the interrelations among dimensions and the probable advantages, disadvantages, and constraints of adopting such novel and disruptive technology.
